# Review of mental health promotion interventions in schools

**DOI:** 10.1007/s00127-018-1530-1

**Published:** 2018-05-11

**Authors:** Michelle O’Reilly, Nadzeya Svirydzenka, Sarah Adams, Nisha Dogra

**Affiliations:** 1 The Greenwood Institute, University of Leicester, Westcotes Drive, Leicester, LE3 0QU UK; 2 Faculty of Health and Life Sciences, De Montfort University, The Gateway, Leicester, LE1 9BG UK; 3 School of Education, University of Leicester, University Road, Leicester, LE1 7RH UK

**Keywords:** Mental health promotion, Children, Schools, Interventions

## Abstract

**Purpose:**

The prevalence of mental disorders amongst children and adolescents is an increasing global problem. Schools have been positioned at the forefront of promoting positive mental health and well-being through implementing evidence-based interventions. The aim of this paper is to review current evidence-based research of mental health promotion interventions in schools and examine the reported effectiveness to identify those interventions that can support current policy and ensure that limited resources are appropriately used.

**Methods:**

The authors reviewed the current state of knowledge on school mental health promotion interventions globally. Two major databases, SCOPUS and ERIC were utilised to capture the social science, health, arts and humanities, and education literature.

**Results:**

Initial searches identified 25 articles reporting on mental health promotion interventions in schools. When mapped against the inclusion and exclusion criteria, 10 studies were included and explored. Three of these were qualitative and seven were quantitative.

**Conclusions:**

A range of interventions have been tested for mental health promotion in schools in the last decade with variable degrees of success. Our review demonstrates that there is still a need for a stronger and broader evidence base in the field of mental health promotion, which should focus on both universal work and targeted approaches to fully address mental health in our young populations.

## Introduction

Globally 10–20% of children and young people experience a mental disorder [28]; and this is increasing [26]. Additionally, it is estimated that 50% of adults with disorders experienced them prior to age 15 [25]. To address this, it is important to pay attention to promotion and prevention practice, with schools being well-placed to deliver. This is because of the amount of time young people spend in this environment [49]. The focus of this review is therefore, on universal mental health promotion interventions in schools rather than those that target high-risk individuals or where health education is part of the treatment of a mental health disorder.

### Mental health promotion and prevention: operational definitions

The World Health Organisation [58] defines mental health promotion as actions to create living conditions and environments that support mental health and allow people to adopt and maintain healthy lifestyles. These include actions to optimise people’s chances of experiencing better mental health. The WHO noted that fundamental to mental health promotion are actions that facilitate an environment that respects and protects basic civil, political, socio-economic and cultural rights. Without the security and freedom provided by these rights, arguably it is difficult to maintain high levels of mental health. The WHO argued that mental health policies should include mental health promotion and not be limited to the health sector, but also involve education, labour, justice, transport, environment, housing, and welfare.

The WHO defines mental illness prevention as encompassing the reduction of incidence, prevalence, and recurrence of illness. Prevention strategies tend to be useful in targeting groups ‘at-risk’ to prevent them from developing disorders. However, although differentiated, it is important to note that the distinction is less rigid for young populations, because children develop skills as they mature [3] and skill development aimed at promoting well-being can have preventative effects [46].

### Mental health promotion in schools

Schools are pervasive environments in young peoples’ lives and can positively impact on their mental health, mitigating some negative impacts of other social factors. However, for some, schools can present as considerable sources of stress, worry, and unhappiness [12], which can hinder academic attainment. In focusing on promotion, therefore, it is important to consider the educational context as a natural environment in which it is possible to build rights of agency, security, and personal freedom in young people, whilst recognising any limitations this may have.

Schools are positioned at the forefront of promoting positive mental health. This is an important way of tackling the growing prevalence of mental disorders worldwide. This has prompted the publication of numerous guidelines and policies in how this could be achieved in the UK and internationally. Recently, in England the government pledged that all secondary (high) schools will receive mental health training by 2020 and each school should have a mental health champion [38]. Similarly, governments in Wales and Scotland have produced policies and statements to advocate the promotion of positive mental health in school-aged children [43, 57]. Furthermore, such thinking is reflected internationally as several countries have been exploring ways of integrating health and education [2].

Evidently, mental health promotion in schools needs to be achieved through the provision of a continuum of intervention programmes. Weist and Murray [55] argued that these should focus on social and emotional learning, competence for all students, and actively involve young people, schools and communities. The authors further argued that quality is central, and many factors need to be accounted for:

- Inclusive approach.
- Build programmes responsive to student, school and community needs, building connections between resources.
- Focus on reducing barriers to student learning through programmes, based on evidence.
- Emphasise and provide support for systematic quality assessment and improvement.
- Ensure staff are engaged and supported.
- Ensure efforts are sensitive to developmental and diversity factors of students.
- Build interdisciplinary relationships in schools, strong teams and coordinating mechanisms.

Weist and Murray [55] observed that for change to happen, training and involvement from a range of people is needed to create a cultural shift in the educational context. This is mirrored in other western countries, where involvement of several people is considered necessary for successful mental health promotion programmes in schools (e.g. [32, 42]). Furthermore, developing partnerships between the health and educational sectors can support meaningful engagement and lasting change [50].

### Whole-school approaches

A ‘whole school approach’ for promoting positive mental health, recognises the importance of working collaboratively with all parts of the school community; students, families and staff, whilst acknowledging the impact of local and government policies [18]. Adopting this approach advocates that schools should tackle mental health and well-being through their behaviour policy, curriculum design, care and support for young people, as well as staff, and engagement of parents. Internationally, this has been implemented through schools adopting social and emotional programmes; for example, in the USA, the Collaborative for Academic, Social, Emotional Learning [8], in Australia, KidsMatter [10] and the UK, Social and Emotional Aspects of Learning (SEAL, DCSF, [9]). Where implemented, it has been found to not only support positive mental health, but also raise academic attainment [37].

Despite the outlined benefits of this approach, it is not without challenges. The whole-school approach advocated by many authors (e.g. [42, 48, 55]), may be undermined by:

- lack of adequate support (in terms of staff willingness and/or funding)
- clarity operationalisation, and consistency in terminology used (this would also need to consider how mental health and illness are conceptualised)
- having appropriately trained staff to provide support and supervision, and
- engaging young people in the development of the promotion of positive mental health.

Furthermore, recognition of the need to have sustainable multi-sector partnership in mental health promotion offers little guidance about who the partnerships should involve or specific roles of stakeholders. However, it would seem appropriate to engage the wider community and include families, as well as young people and their teachers.

### Focus and aims of the review

Research has indicated that many young people worldwide are not well informed about mental health [13, 39, 40, 44, 47], and there is a clear need to raise awareness, educate, and provide interventions that facilitate the maintenance of mental well-being in young populations. Mental health promotions are potentially central to the solution, and therefore, it is unsurprising that many interventions that take this approach have been developed.

The focus of our review is on universal interventions of mental health promotion in schools, recognising that universal and target types require different approaches as the aim of the interventions are different. This review aims to examine advancements in mental health promotion in contemporary education, in the context of global austerity in the last 10 years. In presenting this review, it is necessary to be aware that terminology across the educational and health sectors differs [42] and sometimes mental health promotion is described as positive psychology (e.g. Terjesen et al. [51]) or emotional health (e.g. Kidger et al. [27]). This lack of universal terminology makes reviews complex and comparisons challenging. Therefore, for clarity our searches focused on studies that described interventions as promoting mental health and/or well-being.

## Methods

As noted, the challenge in reviewing mental health promotion is the lack of universality in language and operational definitions of key terms. It is not always clear whether when the term mental health promotion is used, it is consistent with the WHO definition. Additionally, in education, several programmes go under a different title. For example, social and emotional learning (SEAL) is often used and interventions designed to promote effective mastery of social–emotional competencies aim to achieve greater well-being and better school performance by reducing risk factors and promoting protective mechanisms for positive adjustments [20]. For our review, we focused on searching for positive mental health promotion interventions as defined by the WHO, including social and emotional well-being, to capture an inclusive overview of the work that has been done.

### Inclusion and exclusion criteria

To ensure included studies focused on mental health promotion interventions in schools we utilised the literature to facilitate our identification of appropriate inclusion and exclusion criteria. Studies eligible were:

- Written in English.
- Published between 1 January 2007 and 30 November 2017 for three reasons; (1) because there were reviews conducted in the early millennium that captured earlier work (e.g. Wells et al. [56]); (2) a decade is a sufficient time-frame to examine impact and change; and (3) captures recent policy changes that may impact on design and delivery of interventions.
- Universal mental health promotion (or equivalent) (these should be different from targeted approaches as the interventions for universal and targeted interventions have different aims, objectives, intervention type and audience).
- Whole-school interventions, programmes, frameworks, models, and tools, involving many levels of school personnel.
- Target population was school age (that is, children of any age who are attending school. This spectrum varies internationally, but is generally from 3 to 18 years), and included any type of school (e.g. public, private, special, residential).
- Original research.

We also provided parameters by identifying exclusion criteria:

- Not published in English.
- Not book chapters, editorials or guidance documents.
- Not focused on risk factors or related to these.
- Not reporting planning and development, and not pilots of interventions (as these would only present feasibility and would not be conclusive).
- Not those interventions targeting children with pre-existing mental health problems.

### Search strategy

Two large database systems were utilised for the search which captured the multidisciplinary nature of mental health promotion. First, was SCOPUS, a database that captures science, medicine, social science, arts and humanities research. Second, was ERIC, a database of the literature in the field of education. A range of search terms were utilised by two of the authors to ensure the searches were consistent. There were three independent searches across the two databases and these were:

- Mental health AND promotion AND schools
- Positive AND mental AND health AND promotion AND schools AND NOT illness
- Mental health promotion AND well-being AND intervention AND schools

The top 100 results for each key-word combination based on relevance were searched as relevance dropped significantly after this point. This produced 25 articles that appeared to be appropriate. These were mapped against criteria and narrowed to 10 intervention studies.

## Results

When matched against the inclusion and exclusion criteria, a total of ten papers were returned. Three of these utilised a qualitative design and seven quantitative design. The literature was well spread globally (e.g. UK, Australia, USA, Sweden, Denmark, Germany, Ireland) and included different interventions, all of which were targeted at the general population of young people in schools. We organised our findings around four main issues: (1) the theoretical framework underpinning the intervention; (2) support, training and supervision for staff implementing the intervention; (3) outcomes of the interventions and (4) long-term impact. The findings were subsequently summarised and an overview of the articles is presented in Table 1.

**Table 1:** Description of studies

References	Country of research	Methodology and study design	Sample and sample size	Theory	Intervention and its nature	Analysis, outcome and effect size
Neilsen et al. [32]	Denmark	Quantitative—intervention evaluated through pre- and post- intervention questionnaire—given to children, answered during school lesson time A process evaluation was also conducted. The intervention was not considered mature enough for a rigorous design, and thus there was no control group	Children in grades 5–9 aged 11–15 (*N*_t1_ = 589, 53.8% male, 46.2% female; *N*_t2_ = 532, 53.4% male, 46.6% female) Children from two public metropolitan schools Response rate was 96.2% pre-intervention and 83.9% post intervention. Thus, 532 children were included All participants received the intervention	Whole-school approach and Action competence (linking democracy and participation and empowerment) (see Clift and Jensen, [7]). Also, linked whole-school approach to health promoting schools framework	‘Up’—measuring social and emotional competence—promoting mental health using a whole school-approach. Materials were tailored for knowledge, skills, meaning and social action. Aims to reduce socioeconomic inequality in social and emotional competence It has four components; Activities for children; Development of staff skills; Involvement of parents; Initiatives in the everyday life of schools Education materials were tailored to age groups and so they could be integrated into the curriculum Intervention process evaluation was also conducted assessing facilitating factors and barriers to implementation	Contingency tables with Chi-Squared tests (Sig. level 0.05) were used for the analysis. Statistically significant change of children reporting high social and emotional competence from before the intervention (33.3%) to after the intervention (40.8%) No effect size was reported
Franz and Paulus [17]	Germany	Quantitative—pre and post questionnaire + interviews with staff Used a pre–post cohort comparison intervention design (entire cohort received the intervention and was compared to a subsequent cohort without the intervention a year later)	Questionnaires with 32 schools (*N*_teachers_ = 633) (*N*_t1_ = 407, *N*_t2_ = 226) and *N*_students_ = 4019 (*N*_t1_ = 2201, *N*_t2_ = 1818) aged 10–15 years Follow-up 12 months after the intervention	Resource -based conceptual theory, balancing internal and external needs and resources (see Becker, [4])	MindMatters—an Australian programme for mental health promotion in adolescents—encourages respect and tolerance and involves a range of school personnel and children—also encourages resilience The German adaptation involves all involved in the school environment; children, teachers, parents, and so on. It translated the Australian version and parts were culturally adapted It encourages communication and problem-solving Prior to the trial, staff took part in training No intervention fidelity assessment was reported	Authors do not report the group comparison analyses they undertook. Some changes in positive mental health, some improvement in social competence are reported Authors describe effects as minimal (the highest degree of effect (delta) as highest 0.27 for scholastic contribution to social competence)
Kimber et al. [29]	Sweden	Quantitative mixed between-within questionnaire design in RCT setting with 5 yearly assessments	Intervention evaluation focuses on 4 schools—2 SET and 2 non-SET schools (matched in terms of location, SES, demographics, and baseline measures) In SET schools all grades (4–9) received the intervention Sample for evaluation at each time point was aggregated across all groups with the same length of intervention received. Total *N*_SET_ = 1857; total *N*_No−SET_ = 598	No theory discussed	Social and emotional training (SET)—educational techniques This was delivered by class teachers during school hours SET covers, self-awareness, managing emotions, empathy, motivation and social competence Teachers were trained before the intervention and were provided with supervision Intervention fidelity assessment was not reported	Statistical analyses involved: (i) linear regression of each outcome on time in intervention by groups; (ii) Between group effect sizes for DVs (Becker’s delta) (iii) ANOVA/MANOVA for subscales for intervention and non-intervention groups taking into account gender Favourable results over 3 years—(i) Positive outcomes on 5 out of 7 variables—self-report internalising, self-report externalising, mastery, ‘I Think I Am’ and contentment in school. (ii) For the five significant variables, effect sizes were small (0.07) to medium (0.60). (iii) significant interactions between SET and no-SET schools on all but one outcome variables across 5 years (particularly externalising and internalising behaviours) Bullying levels remained consistently low in these schools compared to non-SET schools
Dix et al. [11]	Australia	Quantitative—questionnaires for teachers and parents—Measures of KidsMatter implementation index; socioeconomic, academic performance All students in school received the intervention. Evaluation was conducted on a selected sample in each school. Study lasted 2 years	100 primary(elementary) schools selected form 260 A random stratified sample of children 9.6 (SD = 1.6) years of age with up to 76 students in each school selected. *N*_T1students_ = 4980. Overall 70% response rate for teachers and parents of these students. *N*_teachers_ = 1393 (15.1 years average teaching experience; 85% female). Families were 83% two-parent and 17% one-parent	Whole-school approach; uses a four-part conceptual framework, (1) positive school community, (2) social and emotional learning for children, (3) parent support and education, and (4) early intervention	KidsMatter trial Initiative focused on social and emotional competency Designed to improve mental health, reduce mental health problems, and provide greater support for children with conditions Implementation index was used successfully across participating schools. Schools were categorised into Low, Medium–Low, Medium–High, and High implementing groups and these were accounted for in analysis	Significant results using two-level Hierarchical Linear Modelling analysis of school-level characteristics and academic outcomes. Over 2 years, a 14% shift in teachers’ views that the intervention had led to improvement on academic performance
Fitzpatrick et al. [16]	Ireland	Quantitative—including measures on SDQ, coping strategy checklist, and non-standard measures of help-seeking and ‘what school is like’ Three-year programme A cluster sample-based randomised control trial was used Comparison of standard and enhanced programme	44 primary schools where training had been given to teachers were contacted—31 expressed interest and 17 participated − 1072 students aged 12–16 years took part. Of these, 53% were male, 47% female Stratified for school type, and schools were randomly allocated to standard or enhanced programme	Whole-school approach	Enhanced Social, Personal and Health Education Programme including a mental health component—measured against the standard programme ‘Working things out programme’ based on guidance of children with mental disorders to be used for universal promotion DVD intervention, with support of mental health professionals. On the DVD young people told their stories. These were used to develop the enhanced programme. Intervention fidelity assessment was not reported	A series of factorial ANOVAS looked at effects of time in intervention, condition, gender, and caseness on mental health outcomes. Few differences over time between the two programmes. One statistically significant difference in terms of help-seeking, students showed greater improvement in the enhanced programme in terms of a reduction of peer problems No effect sizes were reported
Anthony and McLean. [1]	Australia	Quantitative—self report surveys to measure protective factors of resilience—completed by children and their class teacher Measures of protective factors, social validity included	39 children aged 8–10 from 2 medium sized suburban primary schools, 15 males, 24 females (mean age, 9.17) 17 were given intervention and 22 control group 1 school was the intervention group, the other a comparison group	Universal approach to promotion	BounceBack intervention—whole school-based resiliency programme, promoting resilience, and positive mental health by teaching social and emotional competencies and positive psychology (see McGrath and Noble, 2003) Intervention enhances protective factors associated with resilience Is a year-long, multi-year programme Consisted of 9 1-h sessions over 9 weeks No training was provided to the teachers or the researchers running the study Intervention fidelity assessment was not reported	Mixed between-within ANOVA assessed change across two time periods in two groups on selected outcomes. One-way repeated measures ANOVA assessed change over time. Overall effectiveness of BounceBack found. Children reported higher levels of resilience post intervention than control (for example, large effect size for recourse index, moderate for vulnerability index). Impact of intervention was maintained at 3-month follow-up
Haraldsson et al. [22]	Sweden	Quantitative—interventional and evaluative pre- and post-test design	Two secondary schools—children aged 12–15 years (6–8 class) Intervention group (school 1) = 153 (90 male, 63 female) Non-intervention (school 2) = 287 (142 male, 145 female) No health promotion had been given previously in these schools Children of similar socio-economic background in each group. Pre- and post-questionnaires were administered at the start and end of academic year	No theoretical framework discussed	Health promotion programme—used as a regular school subject each week for one academic year (25–30 lessons in total). Stress intervention administered by physiotherapist who had experience of stress management Data collected at start and end of school year Intervention fidelity assessment not reported	At baseline no statically significant difference between two groups (using Chi square, *t* test, and Mann–Whitney *U* test). Wilcoxon Signed-rank test was used to compare within groups across times. Those with the stress intervention maintained their sense of wellbeing—non-intervention deteriorated. In both genders No statically significant difference between groups in terms of self-reliance
Butzer et al. [6]	USA	Qualitative (but part of a larger mixed methods) The larger study randomly allocated students to the intervention or control Randomly selected for interview Grounded theory	404 students were enrolled onto the school curriculum Grades 7–12 16 students interviewed, 8 males and 8 females, majority were white Focus of the interviews was on feasibility of yoga and their experience of it. Interviewers were trained in interviewing skills Multiple coders ensured coding reliability	Grounded theory framework—little discussion of theory	Yoga for 7th grade children (aged 12–13 years). Including mindfulness and meditation. Focus on stress management, emotional regulation, confidence building, and promoting peer relationships Yoga sessions were 35 min long and delivered 1–2 times per week It was integrated into the Physical Education curriculum Looked at qualitative differences between phenomenological experiences Intervention fidelity assessment not reported	44% had a positive view of the class, 25% had negative and the rest mixed 69% felt it helped raise mood and manage stress 62% felt it had a positive effect on sleep 25% felt a positive effect on academic performance
Lendrum et al. [31]	United Kingdom	Qualitative—Multiple case study design Interviews and observations which were semi-structured. Interview schedules were informed by the literature and modified in accordance with the approach	National—schools—all 300 secondary schools using the SEAL intervention were invited 48 said yes and 10 were included, but one dropped out, resulting in 9 Schools from 7 local authorities participated to ensure geographical spread Sampling strategy was purposeful	No specific theory identified (but there was some discussion of the whole school approach)	SEAL for adolescents—a social and emotional intervention—whole-school approach—Schools visited once per term over 5 terms Intervention fidelity assessment for SEAL implementation was conducted	Use of NVivo facilitated coding Validity and reliability were supported by triangulation They created a thematic framework. This showed that there was no reported impact on outcomes in social and emotional skills, behaviour or mental health difficulties They showed that there is a need for greater awareness of emotional health and wellbeing in schools. Staff need to be better supported and increase their skills
Hall [21]	United Kingdom	Qualitative—focus groups—educational psychologists working with schools	18 children participated Four focus groups were conducted All children were from reception year (aged 3–5 years) to year 6 (aged 10–11 years) Children were selected by school staff	Child centred framework—children’s rights—creating an environment to foster positive mental health	Ten Element Map—framework and model to promote mental health to identify what works for children in terms of environment, self-esteem, emotional processing, self-management and social participation—used as a tool to elicit children’s perspectives	Positive results, as the framework enabled the identification of children’s mental health skills—whole-school approach effective. A useful framework for accessing children’s voices on the matter Concludes—a useful tool for to promote mental health in schools

### Theoretical frameworks

Most interventions were reported to be underpinned by a theoretical framework, but these were variable. Six studies reported a clear theory underpinning the intervention, and two described the theoretical position of the methodology; two of the studies made no explicit reference to theory. Mostly, studies were underpinned by the framework of a whole-school approach and/or a child-centred approach to mental health promotion [1, 11, 16, 21, 32], although the underpinning theoretical framework was not always clear in the way it was described. Neilsen et al. [32] integrated this whole–school approach framework in the intervention evaluation with an Action Competence focus, linking democracy, participation and empowerment [7]. Franz and Paulus [17] utilised the theoretical position of a resource-based conceptual theory, which balances internal and external needs and resources (see Becker, [4] [non-English publication] in Franze and Paulus, [17]) and did not make explicit reference to the whole-school framework, but did include school personnel in the implementation.

### Support, training, and supervision of staff

A challenge for any intervention is, in part, dependent upon those who deliver it. Notably, seven of the interventions were delivered by teachers, although in one case this was implied rather than stated. Two of the interventions were delivered by specialists including physiotherapists [22] and educational psychologists [21]. For one intervention, the authors did not provide clear details [11]. The support, training, and supervision of teachers during the intervention was described in five of the seven papers that reported staff involvement. For some, training was provided via a workshop [32] and for others, through training sessions. Some staff had continued support and supervision [29], but many did not. Interventions delivered by school staff were also reported to be supported by instruction manuals. Most of these interventions were described as structured [1, 16, 17].

### Mental health outcomes

In reviewing the interventions, all but two clearly reported a positive impact. Eight of the ten interventions highlighted some degree of impact and argued that the intervention was a successful mental health promotion tool. Notably, two interventions did not produce such positive results. Lendrum et al. [31] reported that the national SEAL programme had no significant impact, and this was the case in all schools. They noted that there were several barriers to success, including, challenges and confusion regarding implementation, staff skills and training needs, lack of awareness, reluctance of staff, poor communication and limited coordination of the whole-school approach. Similarly, Fitzpatrick et al. [16] found few differences following the comparison of a standard versus an enhanced intervention programme for mental health promotion. They argued that the difference between the enhanced and the standard programme may be too small to have a statistically significant effect on outcomes.

### Long-term impact

Although most of the interventions demonstrated degrees of success in promoting mental health and well-being, the papers were less clear about the sustainability and maintenance of this success. The eight interventions reporting a positive impact highlighted variability in the long-term outcomes, mostly projecting the potential of the intervention and arguing that long-term evaluations are necessary [1, 17, 32]. Two of the interventions were tested over longer periods of 3- and 2-years [11, 29] respectively, which suggested some sustainability. However, some caution must be exercised as most of the long-term outcomes in terms of mental health promotion were not known and authors argued that commitment from the schools and further evaluations are required in future. Indeed, interventions that showed no change demonstrated that flexibility of the intervention can cause confusion for implementation suggesting the need to balance prescriptive guidelines and flexible adaptations with school culture and ethos [31].

### Overall results

This review has contextualised the broader literature on mental health promotion and specifically explored advancements of universal interventions in the last decade. The results demonstrated that there has been limited advancement of this field. Specifically, we have shown that terminology remains variable, there is still limited evaluation of long-term impacts, and there remains inconsistency regarding the people chosen to run the interventions, with their qualifications and training being varied. Like previous reviews in this area, we demonstrated that methods used were of variable quality, some authors were vague in their descriptions of the intervention, and there was not always clarity regarding sources of funding. Somewhat surprisingly, there was a lack of digital interventions, using AI, informatics, robotics, social media, or internet-based approaches.

## Discussion

Globally, there is continued development and implementation of various interventions in schools designed to promote positive mental health, and yet the effectiveness of most of these is not well evaluated [1]. If we are to move forward and make advances in mental health promotion and help young people cope with daily stresses, we need a better understanding of the outcomes and possible ways of sustaining them. Over the last decade, several mental health promotion interventions have been evaluated and were included in this review.

Universal school-based interventions have great potential to target large populations of young people to promote well-being at a general level. Indeed, this is a common approach taken by schools. Over time, several interventions has emerged based on different theoretical frameworks ([11, 17, 21, 29, 32], to name a few). A unifying factor that often underpins or is central to these universal approaches is the whole-school approach, or at least an approach that requires the cooperation of different levels of school personnel, wider communities, and other agencies. Previous reviews over the last couple of decades on the beneficial effects of mental health, social, emotional and educational outcomes have shown that a whole-school approach sustained for more than a year is positive for health promotion and prevention. These conclusions were supported by Weare and Murray [53] who found that a multi-dimensional and integrated whole-school approach is needed for mental health promotion to be effective and to create positive change in the well-being of young people. A more recent review highlighted that for positive outcomes to be achieved, any intervention must be sequenced in the sense that the activities need to be coordinated, incorporating an active form of learning, focused on personal or social skills and explicitly targeting specific skills rather than positive development [15].

However, these interventions also showcase variability in outcomes, challenges of concepts and ideas, difficulties in implementation and attitudes, and issues of sustainability. Early reviews by Wells and colleagues [56] showed a large variation in type and quality of publications and our review demonstrates that the situation has barely changed since. The quality of evidence has been appraised as generally low-to-moderate, with many studies relying on students’ accounts of their own behaviour, with some studies suffering from high attrition rates [30]. Therefore, while popularity of the universal whole-school approach is undeniable, shortcomings of these interventions need to be addressed. Green et al. [19] stated that “while the limited information from the reviews makes it difficult to comment on universal approaches to mental health promotion, whole-school approaches to the promotion of social and emotional health implemented over years appear to be more effective than brief class-based programmes aimed at preventing mental health problems”. However, like previous reviews, our findings demonstrated that considerable methodological issues remain.

### Challenges of using interventions

The core challenge for successful mental health promotion is that most of the school-based interventions reported tended to be short-term with little long-term follow-up. Furthermore, they were also often evaluated immediately or shortly after the intervention. However, there is increasing evidence that some long-term effects are emerging and that although effects gradually decrease over time they can remain substantial [54].

Although some whole-school approaches related to mental health promotion have found fewer advantages than others, sometimes this is attributed to a lack of consistent, rigorous and faithful implementation of the overall programme and/or lack of support for teachers administering it [29]. For example, in a survey of 599 primary and 137 secondary schools in the UK, two-thirds of schools adopted universal approaches, but gaps in teacher training and support were identified as problematic [52]. For schools with limited resources or those that place high demands on teachers’ time, it may be more beneficial that the universal whole-school approach in the mental health promotion is set aside in favour of a smaller scale targeted intervention that is more manageable and sustainable. The crucial challenge of either model of intervention would be to effectively and consistently engage the learners (that is the young people themselves) in development and delivery.

In attempts to bolster schools’ responsibilities for catering for young peoples’ mental health, funding for schools in England has been provided to ensure all schools have a trained ‘mental health champion’ by 2020 [38]. By having an identified and trained responsible member of staff, this may alleviate some of the challenges faced in implementing a whole-school approach. The ‘mental health champion’ will be able to act as a strategic lead in implementing interventions designed to promote positive mental health, whilst also monitoring the impact and cost effectiveness. However, this raises issues for schools, as training will be central to successful implementation, but training for teachers cannot tackle mental health promotion in isolation from the practical difficulties of supporting children who have diagnosed conditions [41]. Additionally, while training teachers is a positive move to address the large-scale issues, in isolation it will not form the solution as it needs to be part of a continued process supported by greater funding for child mental health [24] otherwise it risks being a “sticking plaster solution” to the challenge [45, n.p]. Currently, the Welsh government is piloting specialist CAHMS workers to act as a link between schools and CAHMS whereby school staff are supported to cater for the mental health of their pupils whilst also having support in place when more specialist interventions are needed [57].

Achieving the goals of mental health promotion, and implementing interventions, relies heavily on good quality evidence, and yet much work in this area is not sufficiently evidence-based [52]. Vostanis et al. argued that there is a clear need to improve this situation. These improvements could include more effective evaluation methodologies (e.g. rationalisation and operationalisation of selected theoretical frameworks and models, methods and instruments used), explicit application procedure of interventions, and details of teacher training and support packages [36]. Evidently, the popularity of a ‘one approach fits all’ needs to be matched with rigorous systematic development, recognising contributing/challenging factors as well as application and measurement across different populations, school systems, and wider cultural contexts. Additionally, more work needs to include the ‘child’s voice’, to be child-centred and respect children’s rights, and therefore, there is a need for more qualitative work in this area.

### Strengths and limitations

This review is not without its limitations. First, to provide a targeted and focused message about mental health promotion in schools, we have been prescriptive in the search terms used to identify the scope of the literature. Given a broader search, we might have included papers that have not utilised specific terminology in their interventions. Additionally, research conducted prior to 2007 was not included, and this work may not have been replicated or evaluated since. These studies were excluded from the review as arguably they may not account for contemporary policy and older reviews may exist which evaluate that work. Second, we have only included results published in English, and therefore, rely on research that has been promoted through English publication streams. While included papers did offer an international perspective in terms of interventions across different educational systems in different countries, the sample remained focused on the developed world. No papers looked at mental health promotion efforts in schools in developing countries, which is an area of great significance in terms of mental health outcomes for young people. This is probably missing from the review due to publishing language barriers and/or research not being undertaken in this area as resources are often even more limited in the developing world. Therefore, the current review and discussion is limited in its applicability to countries with similar development profiles to the ones included. However, arguably, factors of effective and sustainable mental health promotion interventions outlined here could be applicable across variety of cultural contexts, albeit untested.

### Directions for future research

In their review, Weare and Nind [53] identified that the characteristics of high-quality programmes that were successfully implemented include:

- A sound theoretical base with specific, well-defined goals that were communicated effectively.
- Focus on the desired outcomes.
- Explicit guidelines and through training, which is quality assured.
- Complete and accurate implementation.

This list of recommendations is consistent with the need for high-quality training interventions in any field [14]. Weare and Nind [54] argued that much of the evidence related to mental health work in schools would support that these characteristics will be beneficial in implementation, although the benefits may be small and not sustained, as supported by our review findings. The authors, however, argued that even change that is small in statistical terms may translate into a significant impact on well-being and this is something that should be explored.

Nonetheless, it is evident from our review, that there is still a need for a stronger and broader evidence base in the field of mental health promotion, which should focus on both universal work and targeted approaches to fully address mental health in our young populations. In terms of intervention development, research has demonstrated that it is essential to include young peoples’ views when developing interventions to ensure a child-centred approach and support at a whole-school level [21, 35] and thus the co-development of programmes could be helpful. Further to this is the need to develop teachers’ understanding, competence and confidence in delivering and sustaining mental health promotion with their pupils [31], as research shows that teachers are resistant to holding too much responsibility in terms of mental health and lack confidence [34]. Methodologically, interventions need to be able to adapt to school culture and available resources while still offering measurable set of outcomes. More attention needs to be paid to the culture of schools as part of any intervention, as there may be little value in implementing programmes when it is already known that the factors needed for their success are not in place at the time or are not sustainable in long-term (e.g. if funding/support expires with termination of the research project). Furthermore, rigour and quality in the evaluation of interventions also needs attention. Programme effectiveness, safety, and cost is not always as rigorous and robust as it could be [30, 33] and therefore, attention to the quality of studies is essential for future examinations of interventions. Validation tools can go some way to addressing these issues [5].

## Conclusion

Our review has demonstrated that there is some success for interventions, many of which were underpinned by the whole-school approach or similar frameworks. This was also the case for other intervention types that were not so broad in scope. However, training teachers in delivery was important and long-term outcomes unclear. Thus, building on previous work, we have demonstrated that there remain gaps in knowledge, that there are issues with sustainability of universal approaches, and that success, to some extent, relies on cooperation, training and involvement of the schools and the young people themselves. Furthermore, modes of delivery and the nature of the interventions are important and need to appeal to young people. This could be facilitated by more scoping work in terms of digital health promotion. In a digital age, with digital tools, mobile apps, robotics, social media and the internet all forming a central part in daily life, there is potential to integrate a whole-school approach with digital interventions, and there is room to be creative with universal mental health promotion.
